# Development and validation of a risk prediction model for diabetic retinopathy in type 2 diabetic patients

**DOI:** 10.1038/s41598-023-31463-5

**Published:** 2023-03-28

**Authors:** Chengjun Zhu, Jiaxi Zhu, Lei Wang, Shizheng Xiong, Yijian Zou, Jing Huang, Huimin Xie, Wenye Zhang, Huiqun Wu, Yun Liu

**Affiliations:** 1 Department of Medical Informatics, Medical School of Nantong University, Nantong, 226001 Jiangsu China; 2 College of Medical Informatics, Chongqing Medical University, Chongqing, 400016 China; 3 Department of Information, The First Affiliated Hospital, Nanjing Medical University, No.300 Guang Zhou Road, Nanjing, 210029 Jiangsu China

**Keywords:** Endocrinology, Medical research, Risk factors

## Abstract

To establish a risk prediction model and make individualized assessment for the susceptible diabetic retinopathy (DR) population in type 2 diabetic mellitus (T2DM) patients. According to the retrieval strategy, inclusion and exclusion criteria, the relevant meta-analyses on DR risk factors were searched and evaluated. The pooled odds ratio (OR) or relative risk (RR) of each risk factor was obtained and calculated for β coefficients using logistic regression (LR) model. Besides, an electronic patient-reported outcome questionnaire was developed and 60 cases of DR and non-DR T2DM patients were investigated to validate the developed model. Receiver operating characteristic curve (ROC) was drawn to verify the prediction accuracy of the model. After retrieving, eight meta-analyses with a total of 15,654 cases and 12 risk factors associated with the onset of DR in T2DM, including weight loss surgery, myopia, lipid-lowing drugs, intensive glucose control, course of T2DM, glycated hemoglobin (HbA1c), fasting plasma glucose, hypertension, gender, insulin treatment, residence, and smoking were included for LR modeling. These factors, followed by the respective β coefficient was bariatric surgery (− 0.942), myopia (− 0.357), lipid-lowering drug follow-up < 3y (− 0.994), lipid-lowering drug follow-up > 3y (− 0.223), course of T2DM (0.174), HbA1c (0.372), fasting plasma glucose (0.223), insulin therapy (0.688), rural residence (0.199), smoking (− 0.083), hypertension (0.405), male (0.548), intensive glycemic control (− 0.400) with constant term α (− 0.949) in the constructed model. The area under receiver operating characteristic curve (AUC) of the model in the external validation was 0.912. An application was presented as an example of use. In conclusion, the risk prediction model of DR is developed, which makes individualized assessment for the susceptible DR population feasible and needs to be further verified with large sample size application.

## Introduction

Over the past few decades the prevalence of diabetes mellitus (DM) has risen significantly in nearly all countries and may be considered as a growing epidemic^1^. Chronic complications associated with type 2 DM (T2DM) can not only cause a rising burden on the national health system, but also increase the rate of disability, leading to untimely death and reduce the quality of life. In these complications, diabetic retinopathy (DR) is a major retinal disease and a leading cause of blindness in the world^2^.

It is estimated that approximately 600 million people are expected to have diabetes, and one third of them are expected to have DR by 2040^3^. If the patient has a poor control of blood glucose, the long-term high level of blood glucose will pose harm to the body, triggering the likelihood of DR^4^. Besides, though more than half the patients were aware that diabetes could affect the eyes, awareness of DR and its consequences was low^5^. Therefore, the screening of DR, coupled with timely referral and treatment, is a generally accepted strategy for preventing blindness. In order to reduce the risk of DR in patients with diabetes and evaluate its possible risk factors, it is definitely significant to take individualized and targeted interventions.

On account of its considerable public health significance, there have been articles worldwide on DR risk prediction models. In clinical medicine, predictive models refer to the type of medical research that researchers use to try to determine the best combination of medical signs, symptoms, and other findings that can be used to predict the likelihood of a particular disease or outcome^6^. These models can help clinicians make decision regarding clinical admissions, early prevention, early clinical diagnosis, and clinical therapy applications. There are many methods for constructing predictive models of diabetes complications in the current research process, including logistic regression (LR) model, decision tree model, Cox proportional hazard model, back-propagation artificial neural network (BP-ANN) model etc. In a study by Sangi et al.^7^, they applied regression analysis and ANN to create models to explore the association between factors (such as HbA1c, duration, etc.) and DR. A Bayesian belief network was established to show the interaction between all risk factors and DR. In the study of Yao et al.^8^, BP-ANN was used to simulate the relationship between risk factors and DR. Combined with multivariate LR model, the course of diabetes, waist-to-hip ratio, glycated hemoglobin (HbA1c) levels and family history of diabetes are independently associated with DR. However, most datasets were from observational data instead of well-designed clinical trials, which might limit its quality of modeling.

High-quality systematic reviews and meta-analyses are high-quality evidence that synthesize the outcomes from clinical trials^9^. Therefore, we tried to integrate meta-analyses to establish a risk prediction model for DR. In this experiment, we comprehensively searched the databases and collected common risk factors reported for DR, and then selected LR model to develop a risk assessment model for DR incidence in patients with T2DM, thus strengthening the prevention and treatment of DR in T2DM patients.

## Methods

### Workflow of the risk prediction model development

**Figure 1:**
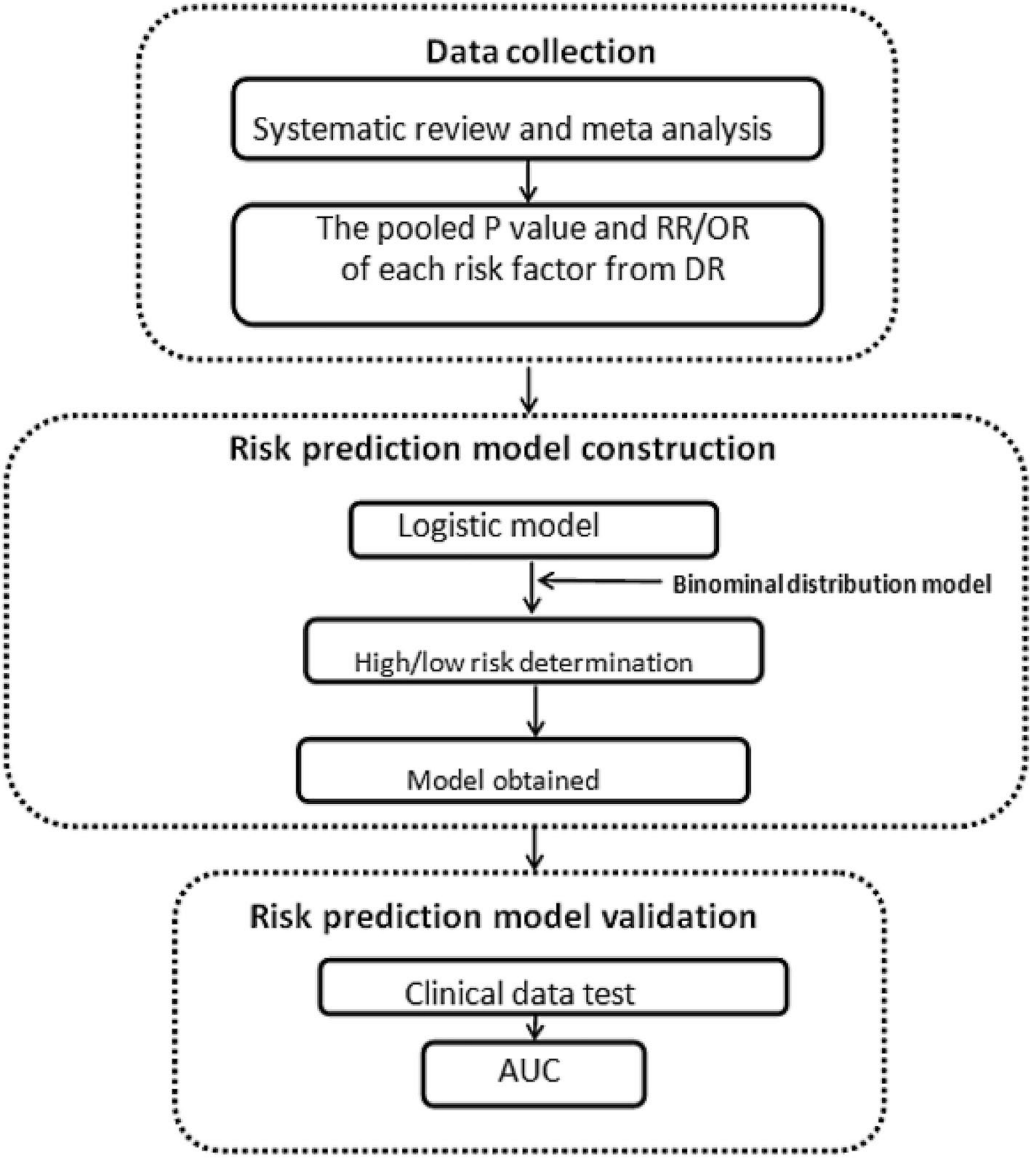
The workflow diagram of this study.

The main contents of this study can be roughly divided into three parts (Fig. 1).

Firstly, relevant studies were retrieved according to the established retrieval strategy and inclusion and exclusion criteria. And data such as the pooled P value and RR/OR of each risk factor from DR vs non-DR were extracted from systematic reviews and meta-analyses. Then the optimized LR model was used to construct the risk prediction model of DR. Based on the binomial distribution data set simulated by Monte Carlo algorithm, the model simulation diagram was drawn, and the high and low risk assessment cut-off value was determined. Finally, the proposed model was verified by clinical questionnaire investigation, and the receiver operating characteristic curve (ROC) was drawn to evaluate its prediction accuracy.

### Search strategy

Comprehensive search for six electronic databases including Chinese National Knowledge Infrastructure(CNKI), WanFang data, PubMed, Web of Science, SpringerLink, VIP were conducted for articles published from the time of the database construction until May 2019. In this study, we focused on the demographics, daily life style, diet, medications etc. as risk factors. The search strategy included the following keywords such as "diabetic retinopathy", "DR", "diabetic macular edema", “DME”, "type 2 diabetes”, "meta-analysis”, “systematic review”, “smoke*”, “drink*”, “weight”, “lipid”, “residence”, “duration”, “glycated hemoglobin”, “HbA1c”, “fasting plasma glucose”, “bariatric surgery”, “myopia”, “hypertension”, “insulin”, “intensive glucose control” and possible combinations. In addition, the Boolean operator AND, OR was used in the search process to combine search keywords in order to obtain full access to all articles. Other electronic databases such as Google Scholar and the Cochrane Library were searched too. We also retrieved grey literatures such as clinical trials and unpublished proceedings, and conducted hand search in those possible paper journals to filter out relevant articles.

### Inclusion criteria

Those meta analyses included should meet the following five criteria. First, the article clearly pointed out the investigated subjects was T2DM patients. Second, risk factors including gender, smoking, residence, smoking, drinking, diet, HbA1c, weight loss surgery, glycemic control, lipid-lowering medications intake duration, insulin usage etc. were included and the importance between those risk factors and DR should be estimators of odds ratio (OR) or relative ratio(RR). In this study, the intensive glycemic control was glucose control until reasonable glucose level was achieved. Course of the disease represented the duration of DM. The HbA1c and fasting plasma glucose was considered as risk factor if the value was abnormal respectively. Third, the DR was defined by internationally accepted diagnostic criteria, according to the 2002 international DR severity grading standard^10^. Fourth, research design was case–control study or cohort study. The case group was patients with DR (the DR group), and the control group was those T2DM patients without DR (the DWR group). Last, the method of data analysis in the literature was correct.

### Exclusion criteria

Those studies were not meta-analyses or the literatures without extractable data. Those studies without OR value and 95% CI or the data provided by the literature cannot be converted to an OR value and a 95% CI value. Studies had no clear case diagnostic criteria. Studies such as reviews, case reports, and animal experiments were excluded. For applicable concerns, those articles not having the included risk factors were excluded. Duplicate published studies, incomplete data reporting, data conflicts, or studies with serious missing data were removed too.

### Study selection

All meta-analyses using the selected keywords prepared a summary list after the search was completed. The requirement for informed consent was waived as this study was designed to extract available data from articles published in peer-reviewed journals and databases. The full text of the article was reviewed, through the reading of the abstract and the full text, the studies were filtered out according to inclusion and exclusion criteria, formulating a basic information extraction table for documents.

### Data extraction

Basic information such as first author’s name, publication year, sample size, age range of included populations, number of cases, study time, type of eye disease were recorded. Furthermore, patient’s demographic data such as age, gender, height, weight, place of residence, as well as risk factors like smoking, bariatric surgery, myopia, lipid-lowering medication intake, fasting plasma glucose, disease duration, HbA1c, glucose control, hypertension, insulin usage were extracted. The importance value between those risk factors and DR, OR and/or RR as well as its 95% CI, were extracted too.

### The prediction modeling with the LR model

During the LR modeling, the risk factors were coded as input variables X_1_, X_2_, X_3_… X_i…_X_n_. Prediction modeling was constructed by fitting a regression model with the attribute variable Xn and its corresponding coefficient βn which could be further calculated with the corresponding Ln (OR).

$${\text{Logit}}\left( {\text{P}} \right) = \upalpha + \upbeta_{{1}} {\text{X}}_{{1}} + \upbeta_{{2}} {\text{X}}_{{2}} + \upbeta_{{3}} {\text{X}}_{{3}} + \cdots + \upbeta_{{\text{i}}} {\text{X}}_{{\text{i}}} + \upbeta_{{\text{n}}} {\text{X}}_{{\text{n}}}$$

where constant α in above formula could be represented by p/(1 − p), which was the prevalence of DR in local population:

$$\alpha = {\text{ Ln}}\left( {\frac{p}{1 - p}} \right)$$

Then, the following regression equations was utilized to estimate patient DR risk:

$${\text{LP}} = \left( {{\text{e}}^{{{\text{Logit}}({\text{P}})}} /\left( {{1} + {\text{ e}}^{{{\text{Logit}}({\text{P}})}} } \right) \, } \right){\text{/p}}$$

where LP was the estimated DR risk of T2DM patient in the local area.

### The determination of high and low risk of DR patients

The 1000 randomly generated data were substituted into the LR model to calculate the probability (P) of DR in diabetic patients. We sorted the P values from small to large, and finally took the sort number ID as the abscissa, the P as the ordinate, and draw a joint distribution of 1000 sample point. R 3.3.1 software was used to calculate and plot joint distribution. According to the change trend of the P, we chose the cut-off value of the P 0.5 as the dividing node of the high and low risk of the DR in T2DM patients (Fig. S1).

### External validation of the proposed DR prediction model

In this study, we established a risk assessment LR model for DR prediction among those T2DM patients. In order to validate the constructed model, we testified its prediction values by investigating patients. An electronic patient-reported outcome questionnaire (Fig. S2) was developed and fulfilled by 60 diabetic inpatients in the Department of Endocrinology of Chongqing Sixth People’s Hospital. Among them, 30 patients had DR diagnosis reported as the case group and the other 30 patients without DR reported were treated as the control group. All the patients completed the questionnaire by themselves or with the consultation to professionals if any uncertainty occurred. After the questionnaires were collected, the data were input into our model and prediction risk results were achieved (Fig. S3).

### Statistical analysis

In order to verify the feasibility of the model we built, we validated the performance of the model by calculating the accuracy, sensitivity, and specificity. ROC was plotted through R script and area under the ROC curve (AUC) was obtained.

### Ethical approval

There are some human participants involved in the current study (android application implementation). This research was performed in accordance with relevant guidelines/regulations and informed consent was obtained from all participants and/or their legal guardians.

## Results

### Search results

**Figure 2:**
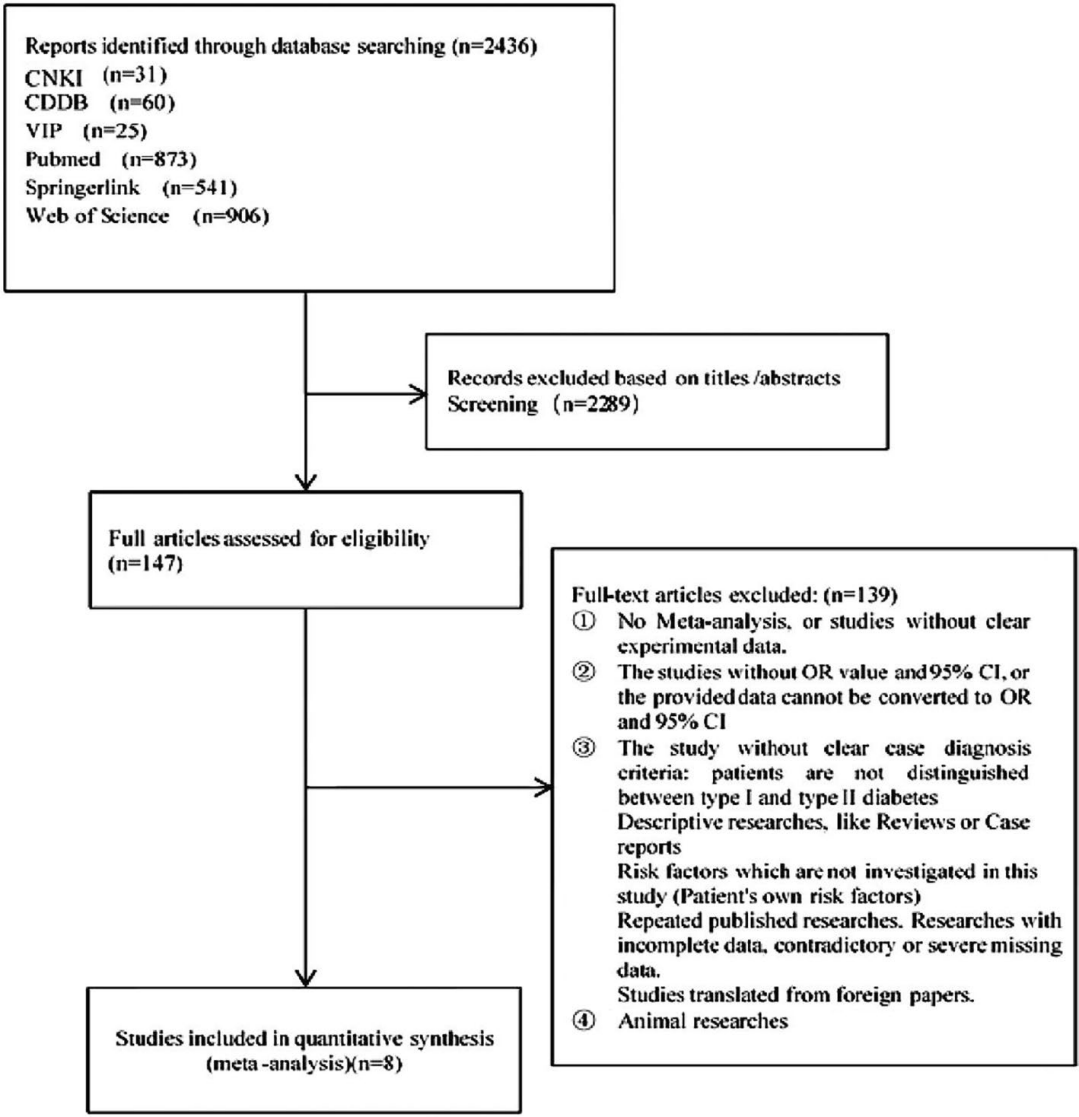
Flow chart of literature search and study selection.

CNKI, WanFang data, PubMed, Web of Science, Springerlink, and VIP were checked. The publication date of the articles was from the period of database construction to May 2019, and a total of 2437 articles on DR were retrieved. We excluded 2290 studies after reading the title and abstract. Another 139 articles including no meta-analysis, no clear experimental data, and no distinction between type 1 and type 2 DM were excluded after reviewing the full text. Finally, eight articles were included. The process of study selection was shown in Fig. 2.

### The characteristics of included risk factors for DR prediction

A total of eight articles^11–18^ were included in the study. The subjects were from all over the world and were older than 18 years old. The final literatures which were included were shown in Table 1.

**Table 1:** The characteristics of the included meta-analysis.

Study	Included studies	DM course (year)	Age range (year)	Sample Size	OR	95%CI	P	Risk factors
Merlotti et al.^11^	7	3.68–14.73	44.62–60.61	NA	0.39	0.21–0.71	NA	Bariatric surgery
Fu et al.^12^	6	NA	NA	4471	0.7	0.58–0.85	NA	Myopia
Shi et al.^13^	2	NA	NA	152	0.37	0.13–1.02	0.05	Lipid-lowering drug (< 3y)
	4	NA	NA	3477	0.8	0.64–1.01	0.06	Lipid-lowering drug (> 3y)
Qiu et al.^14^	11	NA	NA	2890	1.19	1.11–1.27	0	DM course
	7	NA	NA	1720	1.45	1.20–1.75	0.0001	HbA1c
	6	NA	NA	1856	1.25	1.00–1.55	0.05	Fasting plasma glucose
Song et al.^15^	5	NA	NA	NA	1.99	1.34–2.95	0.001	Insulin therapy
	7	NA	NA	NA	1.22	1.10–1.35	< 0.01	Rural residence
Cai et al.^16^	56	NA	NA	NA	0.68	0.86–0.98	< 0.02	Smoking
Liu et al.^17^	141	NA	46.28–70.72	362	1.5	1.20–1.87	NA	Hypertension
	141	NA	46.28–70.72	362	1.73	1.24–2.41	NA	Gender
Zhang et al.^18^	148	NA	NA	364	0.67	0.26–1.73	0.4	Intensive glycemic control

The influencing factors included gender, bariatric surgery, myopia, residence (urban or rural areas), smoking and the course of diabetes, and the combined OR value and its 95% CI was 1.73 (95%CI 0.631–0.722), 0.39 (95%CI 0.21–0.71), 0.7 (95%CI 0.58–0.85), 1.22 (95%CI 1.10–1.35), 0.68 (95%CI 0.86–0.98), 1.19(95%CI 1.11–1.27) respectively. There were two factors of the history of diseases, including high blood pressure and the use time of lipid-lowering drugs (< 3 year, > 3 year). The combined OR value and its 95% CI was 1.50 (95%CI 1.20–1.87), 0.37 (< 3 year, 95%CI 0.13–1.02), and 0.8 (> 3 year, 95%CI 0.64–1.01) respectively. There were four physiological and biochemical indicators, including fasting plasma glucose, HbA1c, intensive glycemic control, and the use of insulin. The combined OR value and its 95% CI was 1.25 (95%CI 1.00–1.55), 1.45 (95%CI 1.20–1.75), 0.67 (95%CI 0.26–1.73), and 1.99 (95%CI 1.34–2.95) respectively.

### A construction of the risk model

In this study, the LR model has been established and X_1_, X_2_ … X_n_ represented gender, bariatric surgery, myopia, lipid-lowering drug use time, fasting plasma glucose, disease course, HbA1c, intensive glycemic control, hypertension, insulin, residence and smoking. βi refers to the Ln (OR) value of each corresponding disease factor was calculated as follows: β_1_ = 0.548, β_2_ = − 0.942, β_3_ = − 0.375. From the subgroup of meta analyses comparing the different period of lipid-lowering drug intake, β_4_ could be given as 0, − 0.994, and − 0.223 for 0 year, less than 3 years, and more than 3 years, β_5_ = 0.223, β_6_ = 0.174, β_7_ = 0.372, β_8_ = − 0.400, β_9_ = 0.405, β_10_ = 0.688, β_11_ = 0.199, β_12_ = − 0.083.

### The performance of the prediction model

**Figure 3:**
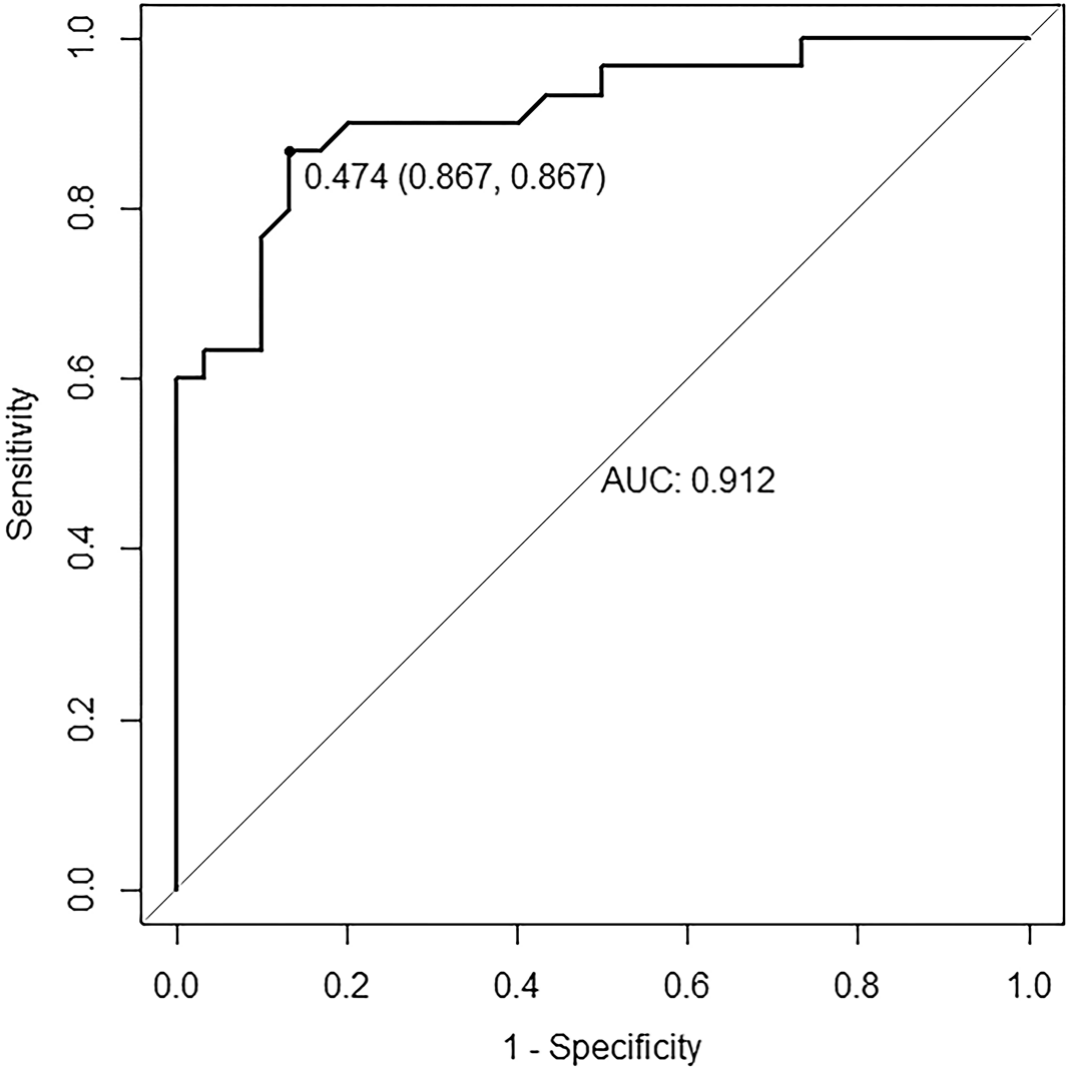
The ROC and AUC of the proposed model.

After the model prediction, DM patients were divided into two risk groups: the high-risk and low-risk group. The high-risk group was set to “DR”, and the low-risk group was defined as “non-DR”. The ROC was obtained using the actual DR data from the clinical investigation and the logistic regression model's prediction results. The verification results of the model were as follows (Fig. 3): AUC was 0.912, the sensitivity value was 0.867 and the specificity value was 0.867.

### An example of use

We prototyped an android application to implement the constructed model in an example use (Fig. S3). Take the prediction process of the risk of developing DR in a 60-year-old male diabetic patient whose personalized information was as follows: height 170 cm, weight 60 kg, no bariatric surgery, no myopia, no lipid-lowering drugs intake, fasting plasma glucose value was 6.5 mmol/L, 10 years of diabetes, HbA1c value was 7%, had intensive glycemic control, high blood pressure, insulin received, living in rural, have a history of smoking. The above features were taken into logit(p) =  − 0.949  +  0.548 + 0.223 + 0.174 + 0.372 −  0.4 + 0.405 + 0.688 + 0.199 −  0.083 = 1.177. Further LP could be calculated by e^1.177^/(1 + e^1.177^)/27.8% ≈ 2.75. The score value represented the patients’ susceptibility to DR than the DWR. Therefore, in this case, the patient was at 2.75 times the risk of developing DR than local diabetic patients from area with DR prevalence of 27.8%. As results indicated, the value greater than 2.24 indicated that the patient was at great risk for DR and the larger risk value was, the higher risk of DR complication that T2DM patients might have. Therefore, those patients with greater predicted value should alter their risk factors or consult a specialist to reduce their risk of developing DR.

## Discussion

DR is the most frequently occurring complication of diabetes and remains a leading cause of vision loss globally.

For the purpose of the patient can evaluate their DR risks by themselves, common life factors were selected for modeling. In this study, based on systematic evaluation and meta-analysis, the risk prediction model of DR in T2DM patients was established by using the optimized LR model with discontinuous categorical variables as dependent features in this study.

In García‐Fiñana’s^19^ study, a multivariate risk model was created to identify patients who would develop sight‐threatening DR. While our study reported the results of the optimized LR model to predict the risk that a given patient with diabetes would develop DR. The model showed high levels of classification accuracy (sensitivity and specificity were 86.7% and 86.7%, respectively). The AUC was 0.912, which was higher than the AUCs previously reported in Scanlon et al.^20^ and Aspelund et al.^21^ of 0.79 and 0.76, respectively. A similar AUC (0.90) was reported in Eleuteri et al.^22^ and although the specificity reported by these authors was 90%, the level of sensitivity was much lower than the sensitivity achieved with our model (67% vs. 86.7%). Other models such as Framingham risk scoring model and Cox proportional hazard model are acceptable, but they still have their own limitations. The risk factors included in the Framingham risk scoring model are relatively fixed. It is difficult to extrapolate to other people with large differences. Also, the Cox proportional hazard model is highly demanding follow-up data^23^. They are commonly used in observational studies^24^ such as case–control studies, cohort studies, etc., to reveal the quantitative relationship between risk factors and outcomes. While the LR model is more flexible, and the coefficients of the model could be appropriately adjusted according to the characteristics of different populations, so that the evaluation model with better prediction effect could be obtained^25^. Compared with machine learning models such as support vector machines, decision trees, artificial neural networks (ANNs)^26^, our DR risk prediction model was interpretable, thus more convenient and intuitive for both patients and clinicians.

There are limited papers about usability of Apps for individual risk assessment for progression of DR compared with those publications on DR prediction by digital retinal imaging^27–29^. We developed a smartphone App which specialized in risk factors that could be perceived by patients in their daily life to assess the progression of DR. By fulfilling the questionnaire in the App, those patients could self-manage their personalized DR risk and update such risk after altering their risk factors, and further consult eye specialist if they were at high risk.

Nevertheless, there are several limitations despite the prediction accuracy of the DR risk prediction model. First, the method of systematic review and meta-analysis was inevitably heterogeneous on account of differences in research design and method as well as characteristics of the cohorts in included studies. However, we can further investigate the source and perform subgroup analysis and sensitivity analysis to reduce the heterogeneity. Second, the risk factors involved in the original literature were not obtained, which might affect the comprehensiveness and reliability of the research. And in the process of research design, data collection and statistical processing, it is inevitable that there are some man-made subjective factors, resulting in deviation. Third, the relationship of the factors and performance of the model is not investigated in this study, and we might take it into consideration for our further research. Therefore, the representativeness and accuracy of the prediction model need to be further verified. Last, DR prevalence may vary across study populations, so a model’s implementation may need to be tailored to each population to improve its performance. Some studies have pointed out that when the exposure rate of the influencing factors was low, the practicability of the model could be optimized and enhanced by simplifying the α of the LR model^30^.

In conclusion, the developed risk prediction model could make individualized assessment for the susceptible DR population to indicate DR hazard ratio and need to be further verified with large sample size application.

## Supplementary Information

Supplementary Figures.

## Data Availability

All data generated or analysed during this study are included in this published article [and its supplementary information files].

## References

[1] Lovic D, Piperidou A, Zografou I (2020). The growing epidemic of diabetes mellitus. Curr. Vasc. Pharmacol..

[2] Ogurtsova K, da Rocha Fernandes JD, Huang Y (2017). IDF Diabetes Atlas: Global estimates for the prevalence of diabetes for 2015 and 2040. Diabetes Res. Clin. Pract..

[3] Ting DSW, Cheung CY, Lim G (2017). Development and validation of a deep learning system for diabetic retinopathy and related eye diseases using retinal images from multiethnic populations with diabetes. JAMA.

[4] Huang YH (2017). Base on the Meta-analysis of Risk of Stroke in Chinese Hypertensive Patients by Risk Appraisal Model.

[5] Rizwan A, Sufyan A, Asghar A (2021). Awareness of diabetic retinopathy among diabetic patients. Pak. Med. Assoc..

[6] McGinn TG, Guyatt GH, Wyer PC (2000). Users' guides to the medical literature: XXII: How to use articles about clinical decision rules. Evidence-Based Medicine Working Group. JAMA.

[7] Sangi M, Win KT, Shirvani F (2015). Applying a novel combination of techniques to develop a predictive model for diabetes complications. PLoS ONE.

[8] Yao L, Zhong Y, Wu J (2019). Multivariable logistic regression and back propagation artificial neural network to predict diabetic retinopathy. Diabetes Metab. Syndr. Obes..

[9] Chalmers TC (1988). Meta-analysis in clinical medicine. Trans. Am. Clin. Climatol. Assoc..

[10] Wilkinson CP, Ferris FL, Klein RE (2003). Global diabetic retinopathy project group. Proposed international clinical diabetic retinopathy and diabetic macular edema disease severity scales. Ophthalmology.

[11] Merlotti C, Ceriani V, Morabito A (2017). Bariatric surgery and diabetic retinopathy: A systematic review and meta-analysis of controlled clinical studies. Obes. Rev..

[12] Fu Y, Geng D, Liu H (2016). Myopia and/or longer axial length are protective against diabetic retinopathy: A meta-analysis. Acta Ophthalmol..

[13] Shi R, Zhao L, Wang F (2018). Effects of lipid-lowering agents on diabetic retinopathy: A meta-analysis and systematic review. Int. J. Ophthalmol..

[14] Qiu L (2007). Meta-analysis of Risk Factors for Type 2 Diabetic Retinopathy.

[15] Song P, Yu J, Chan KY (2018). Prevalence, risk factors and burden of diabetic retinopathy in China: A systematic review and meta-analysis. J. Glob. Health..

[16] Cai XL (2018). The association of smoking and risk of diabetic retinopathy in patients with type 1 and type 2 diabetes: A meta-analysis. Endocrine.

[17] Liu, X. Y. *Logistic Regression Model for Risk Analysis of Complications in Type 2 Diabetes Mellitus Based on Meta-analysis* Chinese (2016).

[18] Zhang X, Zhao J, Zhao T (2015). Effects of intensive glycemic control in ocular complications in patients with type 2 diabetes: A meta-analysis of randomized clinical trials. Endocrine.

[19] García-Fiñana M, Hughes DM, Cheyne CP (2019). Personalized risk-based screening for diabetic retinopathy: A multivariate approach versus the use of stratification rules. Diabetes Obes. Metab..

[20] Scanlon PH, Aldington SJ, Leal J (2015). Development of a cost-effectiveness model for optimization of the screening interval in diabetic retinopathy screening. Health Technol Assess..

[21] Aspelund T, Thornorisdottir O, Olafsdottir E (2011). Individual risk assessment and information technology to optimise screening frequency for diabetic retinopathy. Diabetologia.

[22] Eleuteri A, Fisher AC, Broadbent DM (2017). Individualised variable-interval risk-based screening for sight-threatening diabetic retinopathy: The Liverpool Risk Calculation Engine. Diabetologia.

[23] Barbaresko J, Neuenschwander M, Schwingshackl L (2019). Dietary factors and diabetes-related health outcomes in patients with type 2 diabetes: Protocol for a systematic review and meta-analysis of prospective observational studies. BMJ Open.

[24] Zhou L (2015). Logistic Regression Model for Risk Analysis of Stroke Based on Meta-analysis.

[25] Tsao HY, Chan PY, Su EC (2018). Predicting diabetic retinopathy and identifying interpretable biomedical features using machine learning algorithms. BMC Bioinform..

[26] Stratton IM, Kohner EM, Aldington SJ (2001). UKPDS 50: Risk factors for incidence and progression of retinopathy in type II diabetes over 6 years from diagnosis. Diabetologia.

[27] Tan CH, Kyaw BM, Smith H (2020). Use of smartphones to detect diabetic retinopathy: Scoping review and meta-analysis of diagnostic test accuracy studies. J. Med. Internet Res..

[28] Sheikh A, Bhatti A, Adeyemi O (2021). The utility of smartphone-based artificial intelligence approaches for diabetic retinopathy: A literature review and meta-analysis. J. Curr. Ophthalmol..

[29] Kim TN, Aaberg MT, Li P (2021). Comparison of automated and expert human grading of diabetic retinopathy using smartphone-based retinal photography. Eye.

[30] Christodoulou E, Ma J, Collins GS (2019). A systematic review shows no performance benefit of machine learning over logistic regression for clinical prediction models. J Clin Epidemiol..

