# Supplementary material for: Development and validation of a risk prediction model for diabetic retinopathy in type 2 diabetic patients
Source: Sci Rep. 2023 Mar 28;13:5034. doi: 10.1038/s41598-023-31463-5 (PMC10049996; doi:10.1038/s41598-023-31463-5)
Supplement: Supplementary file 1 — Supplementary Figures. [file 41598_2023_31463_MOESM1_ESM.docx]

Supplementary Material


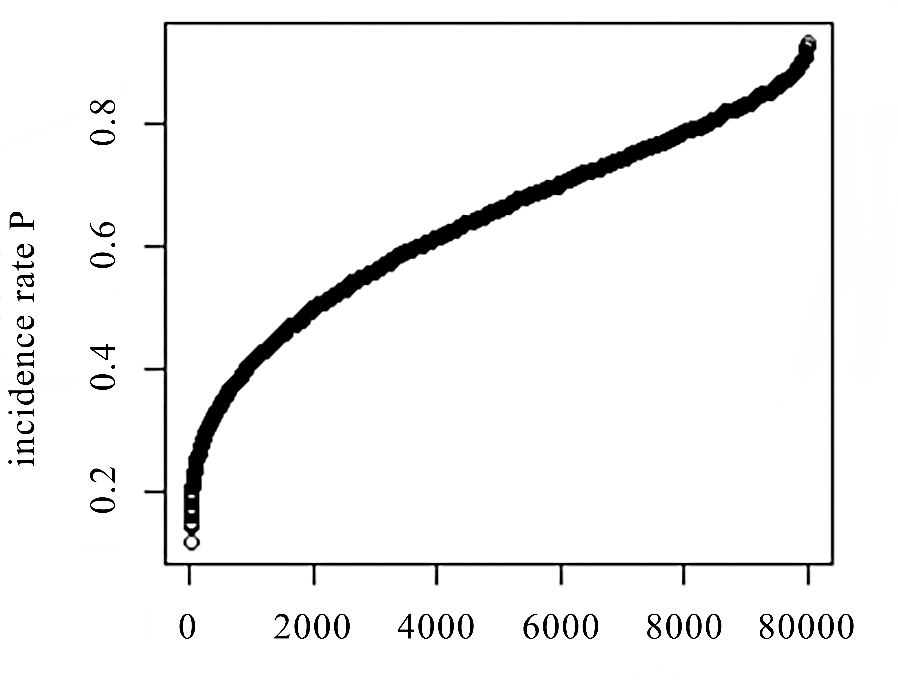


Supplement Figure1 The determination of high and low risk DR patients


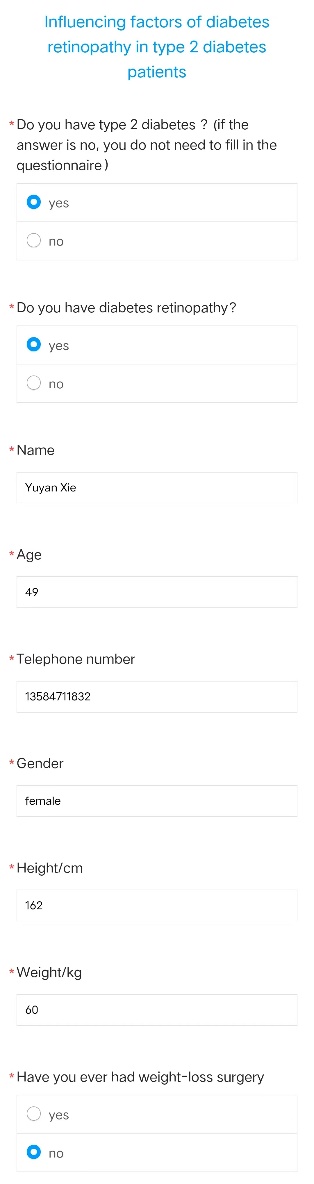

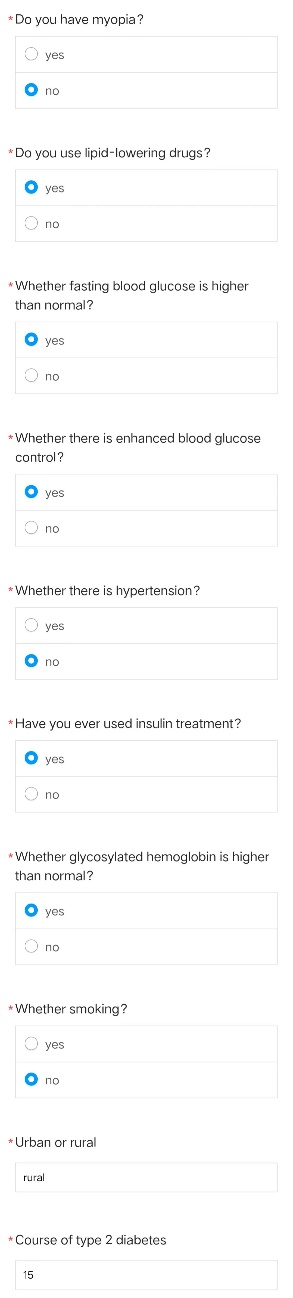


Supplement Figure 2: The screenshot of one questionnaire of the data collection for the validation of our proposed model. Twenty items including the variables for model validation were collected.


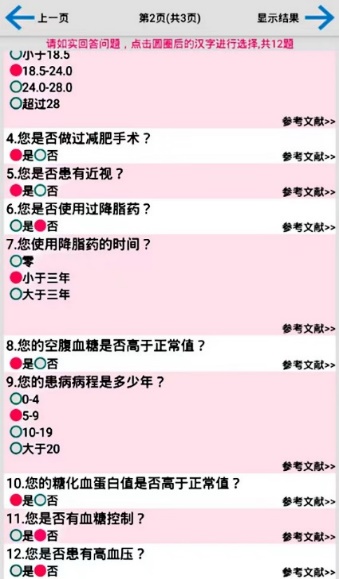


4. Have you ever had weight-loss surgery?

Yes（√） /No

5. Do you have myopia?

Yes （√）/No

6. Have you ever used lipid-lowering drugs?

Yes/No（√）

7. How long have you taken lipid-lowering drugs?

None

Less than 3 years（√）

More than 3 years

8. Is your fasting plasma glucose above normal?

Yes（√）/No

9. How long is your illness?

0-4 years

5-9 years（√）

10-19 years

More than 20 years

10. Is your glycated hemoglobin (HbA1c) higher than the normal value?

Yes（√）/No

11. Do you have blood sugar control?

Yes/No（√）

12. Do you have hypertension?

Yes/No（√）

The results of your option

Choose the second option from the question 1. The ln (OR) represented by this option is equal to 0.548.

Choose the first option from the question 2. The ln (OR) represented by this option is equal to 0.0.

Choose the third option from the question 3. The ln (OR) represented by this option is equal to 0.0.

Choose the second option from the question 4. The ln (OR) represented by this option is equal to 0.0.

Choose the first option from the question 5. The ln (OR) represented by this option is equal to -0.357.

Choose the second option from the question 6. The ln (OR) represented by this option is equal to 0.0.

Choose the first option from the question 7. The ln (OR) represented by this option is equal to 0.0.

Choose the first option from the question 8. The ln (OR) represented by this option is equal to 0.0.

Choose the first option from the question 9. The ln (OR) represented by this option is equal to 0.0.

Choose the second option from the question 10. The ln (OR) represented by this option is equal to 0.372.

Choose the second option from the question 11. The ln (OR) represented by this option is equal to 0.0.

Choose the second option from the question 12. The ln (OR) represented by this option is equal to 0.0.

The sum of the total ln (OR) values is equal to 0.5630000000000001.

The corresponding logit (P) value is equal to -1.174.

The risk value (P) of diabetes eye disease is equal to 1.5742181777821214.


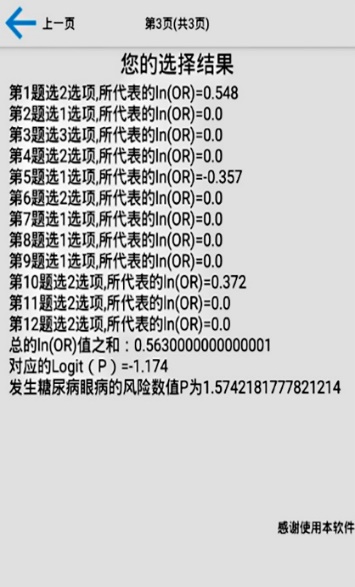


Supplement Figure 3 Android application for validation based on the model

As the interface showed (the right panel is the English version of the original one), the application set the risk factors as variables of questionnaire. After the options for each patient was selected, the constructed model using the input variables and the risk value of the patient had DR was calculated. The result page will demonstrated the potential risk of DR compared with the baseline T2DM population.
